# Isolation of a Glucosamine Binding Leguminous Lectin with Mitogenic Activity towards Splenocytes and Anti-Proliferative Activity towards Tumor Cells

**DOI:** 10.1371/journal.pone.0038961

**Published:** 2012-06-14

**Authors:** Yau Sang Chan, Jack Ho Wong, Evandro Fei Fang, Wenliang Pan, Tzi Bun Ng

**Affiliations:** 1 School of Biomedical Sciences, The Chinese University of Hong Kong, Hong Kong; 2 Laboratory of Molecular Gerontology, National Institute on Aging, Baltimore, Maryland, United States of America; University of South Florida College of Medicine, United States of America

## Abstract

A dimeric 64-kDa glucosamine-specific lectin was purified from seeds of *Phaseolus vulgaris* cv. “brown kidney bean.” The simple 2-step purification protocol involved affinity chromatography on Affi-gel blue gel and gel filtration by FPLC on Superdex 75. The lectin was absorbed on Affi-gel blue gel and desorbed using 1M NaCl in the starting buffer. Gel filtration on Superdex 75 yielded a major absorbance peak that gave a single 32-kDa band in SDS-PAGE. Hemagglutinating activity was completely preserved when the ambient temperature was in the range of 20°C–60°C. However, drastic reduction of the activity occurred at temperatures above 65°C. Full hemagglutinating activity of the lectin was observed at an ambient pH of 3 to 12. About 50% activity remained at pH 0–2, and only residual activity was observed at pH 13–14. Hemagglutinating activity of the lectin was inhibited by glucosamine. The brown kidney bean lectin elicited maximum mitogenic activity toward murine splenocytes at 2.5 µM. The mitogenic activity was nearly completely eliminated in the presence of 250 mM glucosamine. The lectin also increased mRNA expression of the cytokines IL-2, TNF-α and IFN-γ. The lectin exhibited antiproliferative activity toward human breast cancer (MCF7) cells, hepatoma (HepG2) cells and nasopharyngeal carcinoma (CNE1 and CNE2) cells with IC_50_ of 5.12 µM, 32.85 µM, 3.12 µM and 40.12 µM respectively after treatment for 24 hours. Flow cytometry with Annexin V and propidum iodide staining indicated apoptosis of MCF7 cells. Hoechst 33342 staining also indicated formation of apoptotic bodies in MCF7 cells after exposure to brown kidney bean lectin. Western blotting revealed that the lectin-induced apoptosis involved ER stress and unfolded protein response.

## Introduction

Lectins are sugar binding proteins or glycoproteins that consist of one or more binding sites for interaction with their specific carbohydrates. Lectins can be found in various types of plants [1]–[3]. Plant lectins exhibit a variety of biological activities like anti-bacterial [4], anti-viral [5], anti-fungal [2] and anti-insect [3] activities. The anti-pathogenic activities of plant lectins enable themselves to act as defense proteins to protect the plants from invasion of harmful organisms. Other than for the plants’ own uses, the plant lectins also exhibit other biological activities, such as inducing mitogenic response in mammalian splenocytes [1], [2], [6] and anti-proliferative effects toward tumor cells [7], [8]. These activities provide insights for plant lectins to be applied to humans for therapeutic uses, such as development of immunomodulatory drugs or anti-tumor drugs. For example, mistletoe lectin from *Viscum album* exhibited immunostimulatory and cytotoxic effects while having a low toxicity [9], [10], allowing it to be used for treatment of cancers [11].

*Phaseolus vulgaris*, also known as common bean, is a leguminous species producing edible beans. There are many different cultivars of common beans, with most of their beans consisting a lectin or a phytohemagglutinin as one of the major storage proteins. Those lectins have similar molecular sizes (around 60 kDa), but many of them exhibit different biological activities. For example, the lectin from French bean cultivar no. 1 induced mitogenic response in murine splenocytes [12] but not French bean Indian cultivar lectin [13]. Lectin from French bean cultivar no. 35, but not that from French bean cultivar no. 1 inhibited the growth of MCF7 breast cancer cells and HepG2 hepatoma cells [12], [14]. *Phaseolus* lectins possessing anti-tumor activities show different potencies toward different tumor cells. Lectin from French bean cultivar no. 35 had potent anti-proliferative activity on MCF7 cells and weaker activity toward HepG2 cells [14], while lectin from blue tiger king bean had specific anti-proliferative activity toward HepG2 cells [15]. On the other hand, several *Phaseolus* lectins had anti-tumor activity on colon cancer cell lines like Caco-2 cells [16], [17], while some other *Phaseolus* lectins were inhibitory cervical cancer HeLa cells [18]. However, lectin from tepary bean could inhibit viability of both colon cancer Sw480 cells and cervical cancer C33-A cells [19]. As different *P. vulgaris* lectins possess different biological activities, investigation of new *P. vulgaris* lectins would provide chances for identification of lectins with good potential for therapeutic uses.

**Table 1:** Sequences of the upper and lower primers used in PCR.

Cytokine	Primer	Sequence (5′ to 3′)	Size (b. p.)
IL-2	Upper	TTGATGGACCTACAGGAGCTCCTGAGCA	393
	Lower	AGAGAGCCTTATGTGGTTGTAAGCAGGAGG	
IFN-γ	Upper	AGGAACTGGCAAAGGATGGTG	353
	Lower	GTGCTGGCAGAATTATTCTTATTG	
TNF-α	Upper	TCCCCAAAGGGATGAGAAGTTC	411
	Lower	TCATACCAGGGTTTGAGCTCAG	
GAPDH	Upper	ACCACAGTCCATGCCATCAC	452
	Lower	TCCACCACCCTGTTGCTGTA	

In the present study, we have purified a lectin from brown kidney beans (*P. vulgaris*) with two simple chromatographic steps. Some physical and chemical properties such as thermostability, pH stability and carbohydrate specificity of the lectin have been investigated. Biological activities of brown kidney bean lectin were also studied. The lectin was found to exhibit immunomodulatory effects by evoking mitogenic response and inducing cytokine production in murine splenocytes. Besides, the lectin induced anti-proliferative effects on some tumor cell lines by inducing unfolded protein response and apoptosis. The immunostimulatory effect and anti-tumor effects of brown kidney bean lectin suggested therapeutic potentials of the lectin.

**Figure 1:**
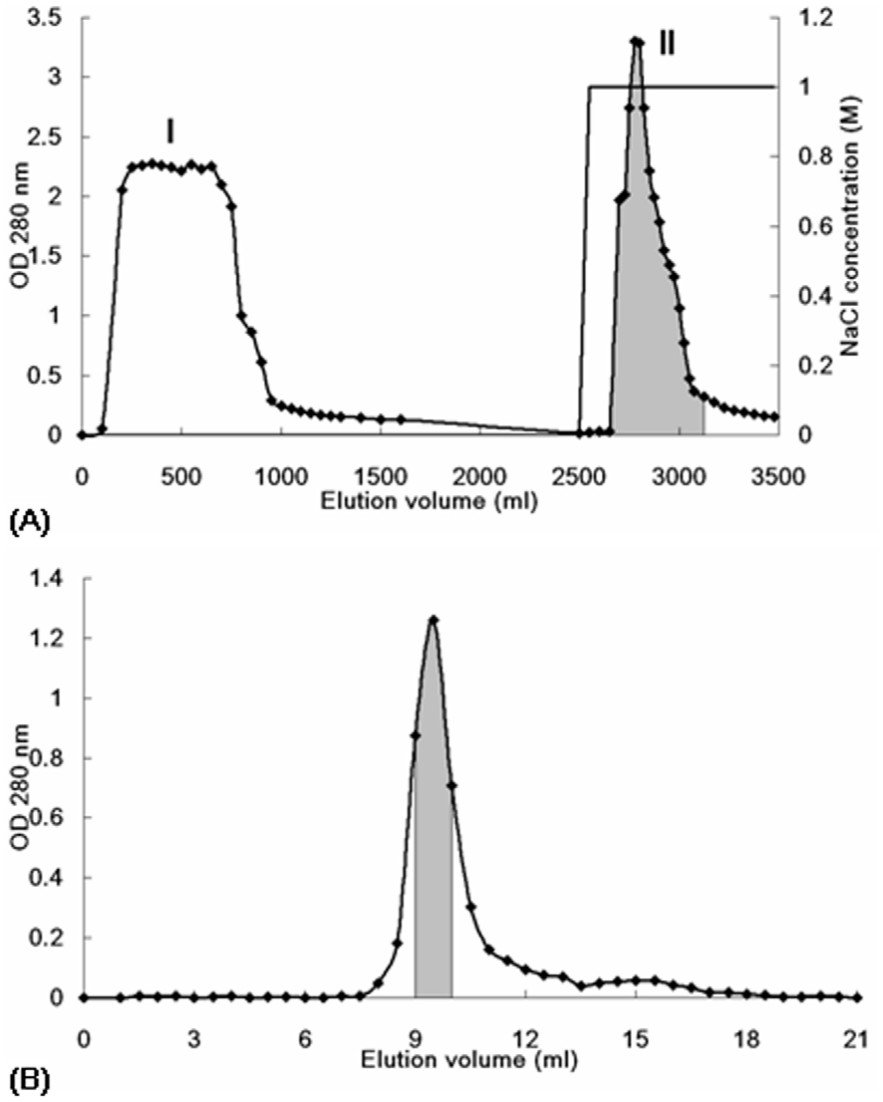
Profile of elution of bean extract from (A) Affi-gel blue gel and (B) Superdex 75.

**Figure 2:**
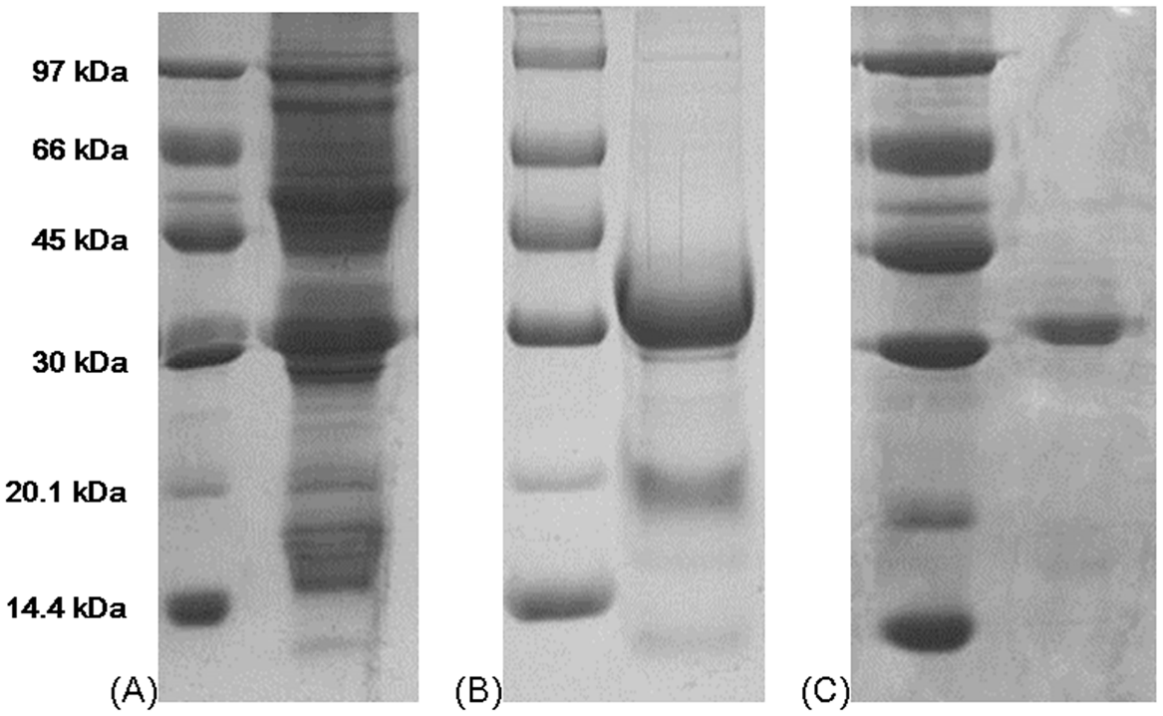
Results of SDS-PAGE. SDS-PAGE of (A) crude extract of brown kidney beans, (B) fraction bound to Affi-gel blue gel and (C) purified brown kidney bean lectin from Superdex 75, respectively. A single 32-kDa band corresponding to purified brown-kidney bean lectin was obtained after FPLC-gel filtration on Superdex 75. The left lane in each of the panels A, B and C shows the molecular mass marker proteins.

**Table 2:** Table of purification of brown kidney bean lectin.

Step of purification	Yield (mg)/60 g beans	Specific hemagglutinating activity (units/mg)	Total hemagglutinating activity (units)	Recovery of hemagglutinatingactivity (%)	Fold of purification
Crude extract	9000	1638	14745600	100	1
Affi-gel blue gel	477	21845	10420224	70.67	13.3
Superdex 75	257	32649	8395200	56.93	19.9

## Materials and Methods

### Purification of Brown Kidney Bean Lectin

The seeds of *Phaseolus vulgaris* cv. “brown kidney bean” were a product of Mainland China. The seeds were extracted in distilled water (10 ml/g) using a Waring blender, followed by centrifugation twice at 30000 *g*, 4°C, 30 minutes. The supernatant was adjusted to 10 mM Tris-HCl, (pH 7.6) by adding Tris-HCl buffer (2 M, pH 7.6). The supernatant was loaded onto an Affi-gel blue gel (Bio-Rad) column (18 cm×5 cm) that has been equilibrated with 10 mM Tris-HCl buffer (pH 7.6). Unadsorbed proteins were eluted with the starting buffer. The column was washed with 1 M NaCl in 10 mM Tris-HCl buffer to elute the adsorbed proteins. The adsorbed fraction was dialyzed extensively against double distilled water overnight at 4°C. The dialyzed fraction was lyophilized into powder form, and resuspended in distilled water (10 mg/ml). The solution was subjected to FPLC-gel filtration on a Superdex 75 10/300 GL column (GE Healthcare) using an AKTA Purifier (GE Healthcare). A major absorbance peak that contained purified brown kidney bean lectin was detected.

**Figure 3:**
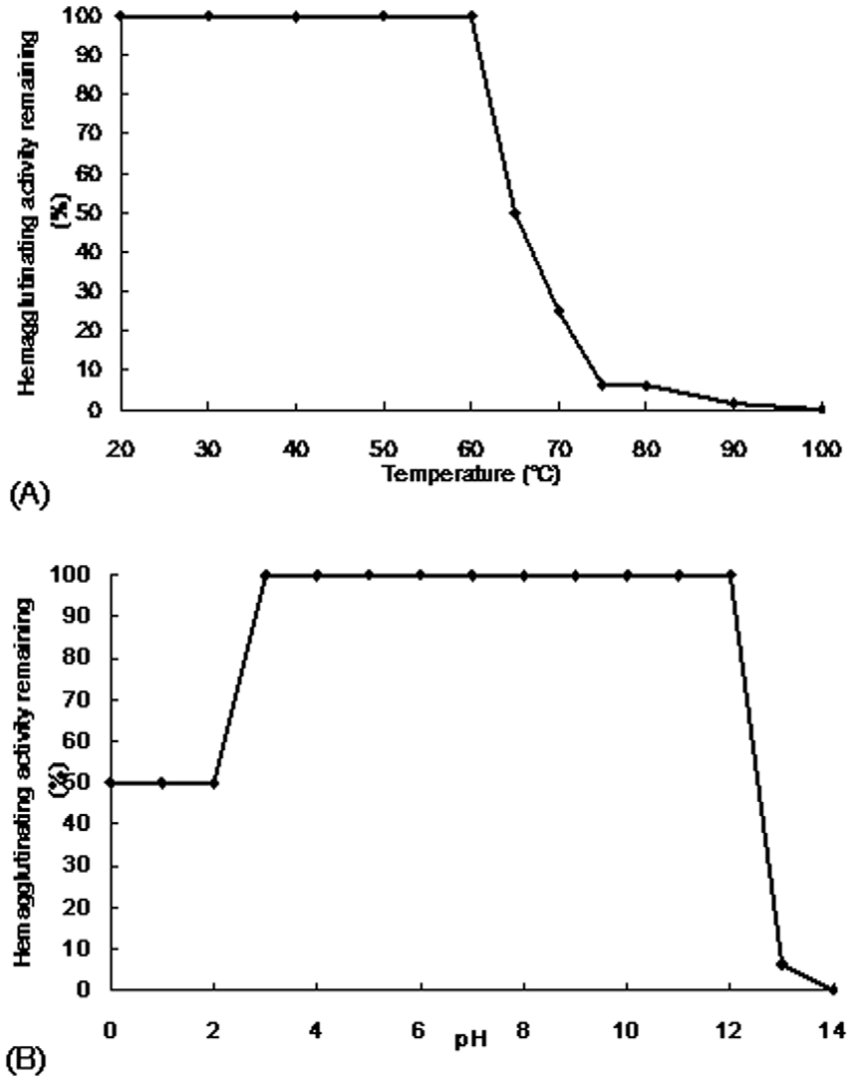
Effect of (A) temperature and (B) pH on hemagglutinating activity of brown kidney bean lectin. Brown kidney bean lectin was stable up to 60°C, and in the range of pH 3 to 12.

**Table 3:** Effects of different carbohydrates on hemagglutinating activity of brown kidney bean lectin (BKBL).

Carbohydrate	Hemagglutinating activityremaining (%)	[Glucosamine](mM)	Hemagglutinating activityremaining (%)
Glucose	100	0	100
Mannose	100	6.25	100
Galactose	100	12.5	50
Lactose	100	25	50
Glucosamine	25	50	50
Glucuronic acid	100	100	50
Galactonic acid	100	150	25
Xylose	100	200	25
Xylitol	100	250	25
Raffinose	100		
Mannitol	100		
Arabinose	100		

### Assay of Hemagglutinating Activity

In a 96-well microtiter U-plate, a serial two-fold dilution of the test sample (50 µl) in phosphate-buffered saline (PBS) (pH 7.2) was preformed. A 2% rabbit red blood cell suspension (50 µl) (purchased from Laboratory Animal Service Center, The Chinese University of Hong Kong) in PBS was added to the sample. The mixture was incubated at room temperature until the red blood cells in the blank (no protein sample) had fully sedimented and appeared as a red spot at the bottom of the well. Presence of agglutinated red blood cells in the wells indicated hemagglutinating activity. One hemagglutination unit (hemagglutination titer) is the reciprocal of the highest dilution of the lectin sample inducing hemagglutination. Specific activity is the number of hemagglutination units per mg protein [20].

**Table 4:** N-terminal sequence of brown kidney bean lectin, compared with lectins of other *P. vulgaris* cultivars and other *Phaseolus* species.

Species of the lectin	N-terminalsequence	% identity
Brown kidney bean (*P. vulgaris*)	^1^ANEEYFDFQQ^10^	100
French bean cultivar no. 1 (*P. vulgaris*)	^1^A**T**E**N**YF**S**FQ**R** ^10^	60
French bean cultivar no. 12 (*P. vulgaris*)	^1^A**T**E**T**YF**N**FQ**R** ^10^	60
Hokkaido red bean (*P. vulgaris*)	^1^AN**TS**YF**N**FQ**R** ^10^	60
Pinto bean (*P. vulgaris*)	^1^A**S**E**TS**F**S**FQ**R** ^10^	50
*P. acutifolius*	^25^AN**DIS**F**N**FQ**R** ^34^	50
*P. glabellus*	^25^AN**DIS**F**N**F**DT** ^34^	40
*P. costaricensis*	^22^A**S**E**TS**F**S**F**DR** ^31^	40
*P. coccineus*	^6^A**S**E**TS**F**S**F**DR** ^15^	40
*P. maculatus*	^25^ **T**N**LFS**F**N**FQ**K** ^34^	40
*P. lunatus*	^25^AN**DIF**F**NIDT** ^34^	30

Amino acid residues different from those in BKBL are bolded.

### Molecular Mass Determination

Sodium dodecyl sulfate-polyacrylamide gel electrophoresis (SDS-PAGE) with a 15% separating gel and a 5% stacking gel was performed at a constant voltage of 120 V. After electrophoresis, the gel was stained with Coomassie brilliant blue with shaking using a shaker for 1 hour, and destained with 10% acetic acid overnight [21].

**Figure 4:**
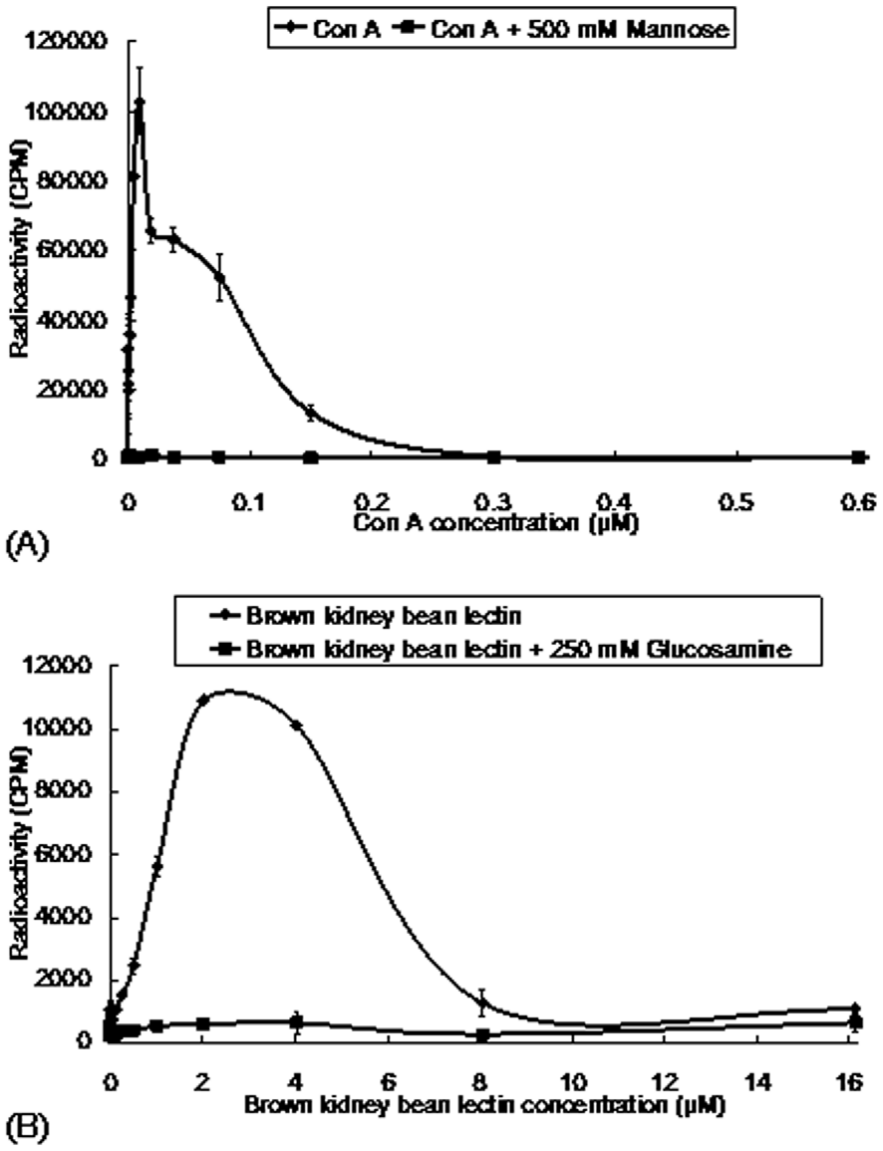
Mitogenic response of murine splenocytes induced by (A) concanavalin A (Con A) and (B) brown kidney bean lectin. Con A acted as the positive control, and its mitogenic activity was blocked in the presence of mannose. Brown kidney bean lectin exhibited maximal mitogenic activity on murine splenocytes at 2.5 µM. Its activity was attenuated by glucosamine. Data represent mean±SD (n = 3).

**Figure 5:**
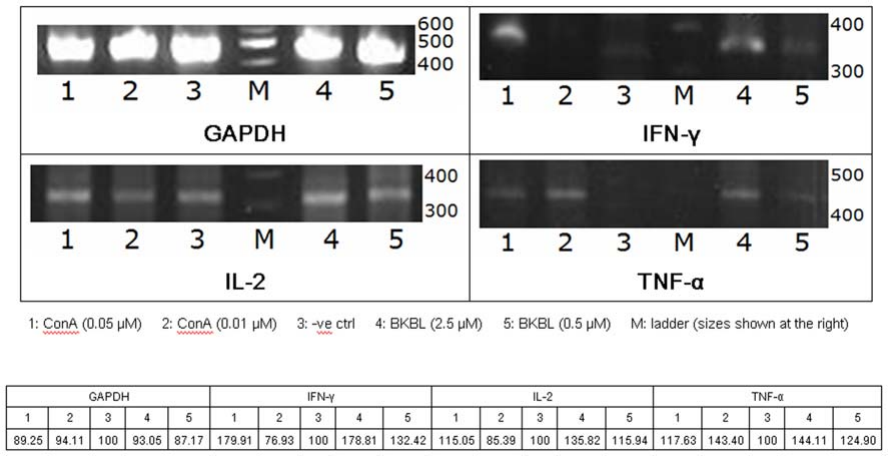
Induction of cytokine gene expression in mouse splenocytes by brown kidney bean lectin (BKBL) and concanavalin A. Brown kidney bean lectin was found to induce the expression of IL-2, IL-1β, IFN-γ and TNF-α at 2.5 µM. The results have been quantified by the software ImageJ. The relative intensities of lanes 1, 2, 4 and 5 for gene expression of each cytokine are shown in the table above.

FPLC-gel filtration was carried out using a Superdex 75 10/300 GL column (GE Healthcare) previously calibrated with molecular-mass standards [22].

**Figure 6:**
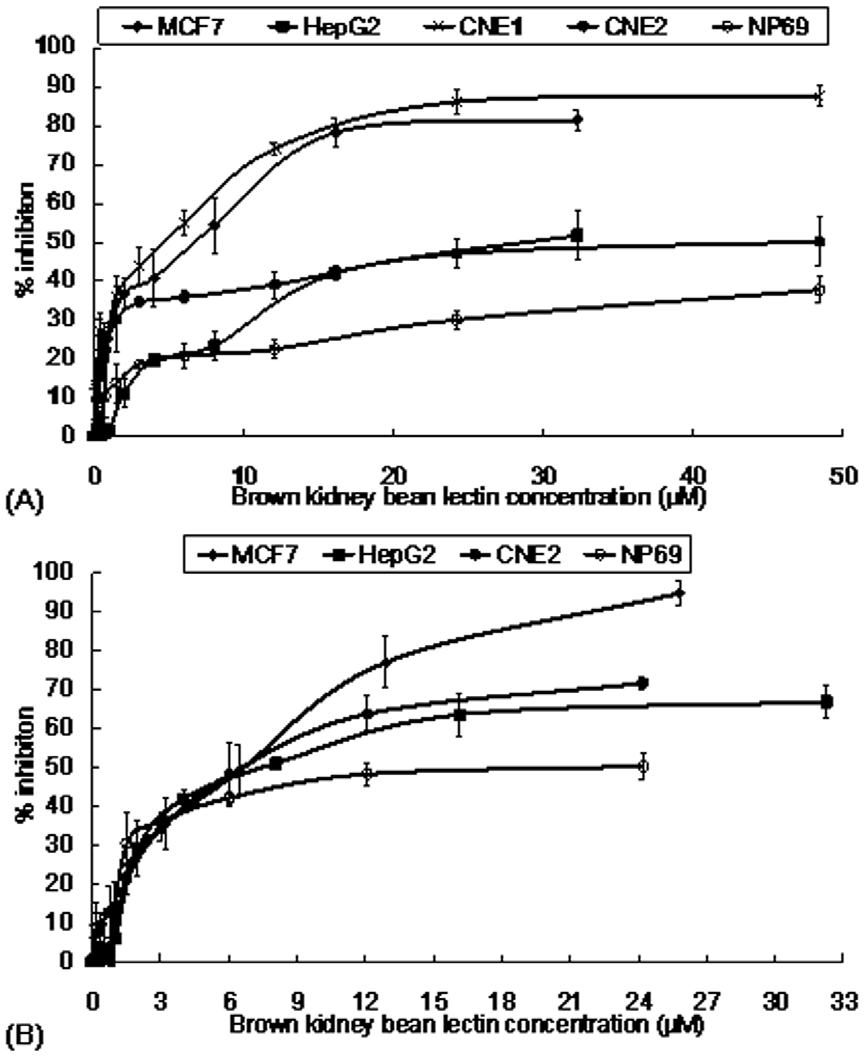
Results of MTT assay on different cell lines. The cells were treated with brown kidney bean lectin for (A) 24 hrs and (B) 48 hrs. Brown kidney bean lectin exerted strong anti-proliferative activity on MCF7 and CNE1 cells, mild anti-proliferative activity on HepG2 and CNE2 cells and weak anti-proliferative activity on NP69 cells. Results represent mean±SD (n = 3).

### Protein Concentration Determination

The protein concentration was determined using the Bradford reagent. Bovine serum albumin was used as the standard [23].

**Figure 7:**
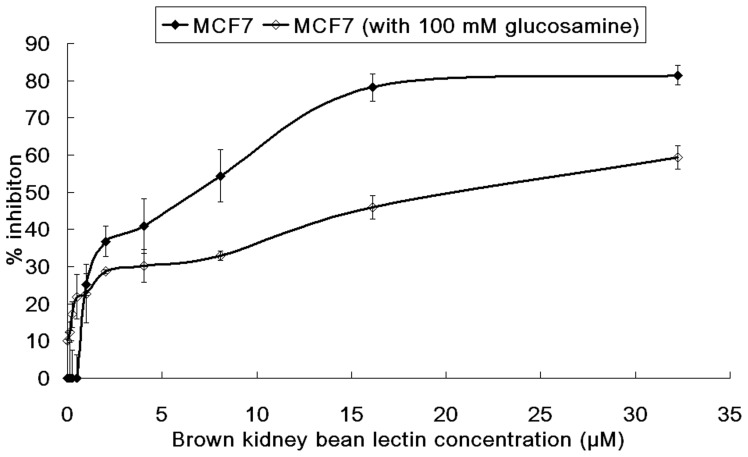
Results of MTT assay on MCF7 cells with glucosamine. The cells were treated with brown kidney bean lectin in the presence or absence of 100 mM glucosamine. Addition of 100 mM glucosamine had reduced the anti-proliferative activity of brown kidney bean lectin on MCF7 cells. Results represent mean±SD (n = 3).

### N-terminal Sequencing

Analysis of N-terminal amino acid sequence was performed by automated Edman degradation, using a Hewlett Packard 1000A protein sequencer equipped with an HPLC system [24].

**Figure 8:**
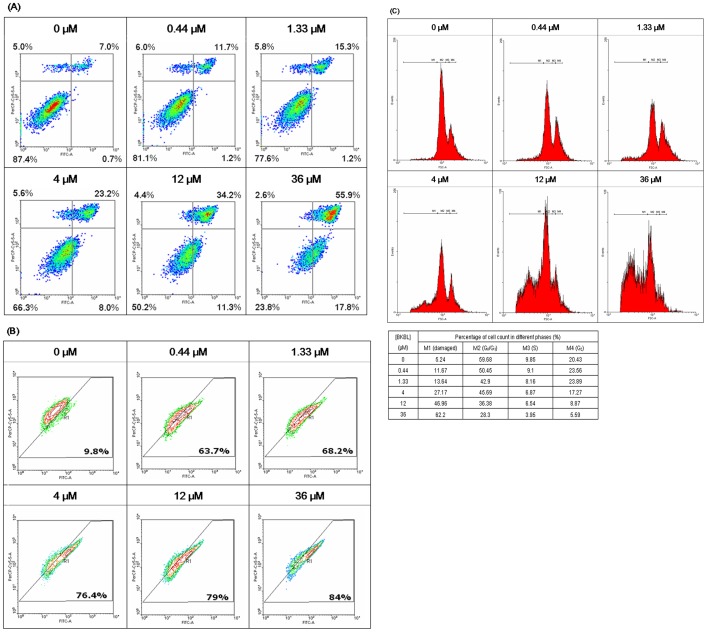
Flow cytometry analysis. Flow cytometry of (A) Annexin V-PI staining, (B) JC-1 staining and (C) cell cycle analysis on MCF7 cells treated with different concentrations of brown kidney bean lectin (BKBL) for 24 hrs. WinMDI 2.9 was used for data analysis.

### Effect of Temperature, pH and Carbohydrates on Lectin-induced Hemagglutination

To test the effect of temperature, the protein sample was heated at 20°C to 100°C for 30 minutes. The sample was immediately cooled on ice to terminate the incubation and then allowed to warm up to room temperature. The hemagglutinating activity assay was performed. Percentage of hemagglutinating activity was calculated by dividing the activity of the sample by the activity of that at room temperature × 100% [12].

**Figure 9:**
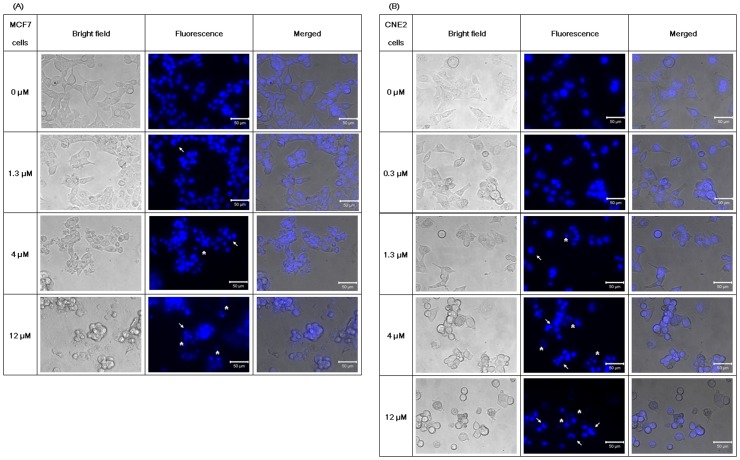
Hoechst 33342 staining. Fluorescence microscope images on Hoechst 33342 staining from (A) MCF7 cells and (B) CNE2 cells treated with brown kidney bean lectin for 24 and 48 hours respectively. Morphological changes of the cells (shrinking, dying) upon brown kidney bean lectin treatment were observed in the bright field. Formation of apoptotic bodies (indicated by asterisks) and chromatin condensation (indicated by arrows) was visualized by the fluorescence of Hoechst 33342 labeling.

To test the effect of pH, solutions of pH 0 to 14 were prepared using the following reagents: pH 0–1: HCl; pH 2–5: NH_4_OAc; pH 6–10: Tris-HCl; pH 11–12: NaHCO_3;_ and pH 13–14: NaOH. Equal volumes of protein sample and pH solution were mixed and incubated at room temperature for 30 minutes. The sample was neutralized and the hemagglutinating activity assay was performed. Percentage of hemagglutinating activity was calculated [25].

**Figure 10:**
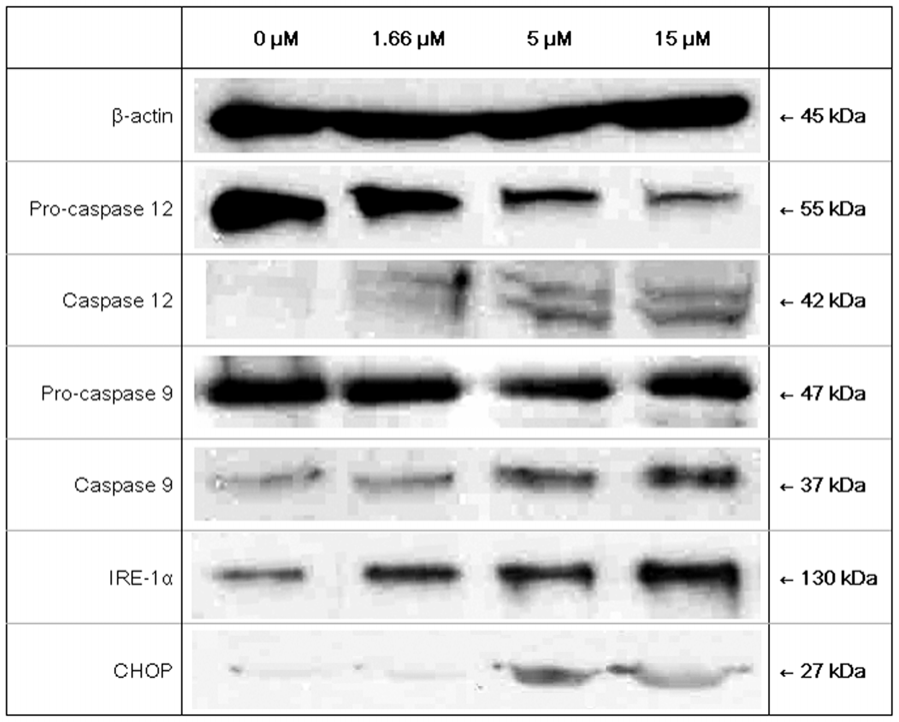
Results of Western blotting. Western blotting of MCF7 cells with 24-hour treatment of 0 µM, 1.66 µM, 5 µM and 15 µM brown kidney bean lectin. The lectin treatment had resulted in activation of caspase 12 (reduction in pro-caspase 12), caspase 9, and up-regulation of IRE-1α and CHOP.

To test the effect of carbohydrates, brown kidney bean lectin was dissolved in solutions of different carbohydrates (500 mM) in PBS. The hemagglutinating activity assay was performed as described before, but the 500 mM carbohydrate solutions were used for serial two-fold dilution instead of PBS. Percentage of hemagglutinating activity was calculated. Reduction in hemagglutinating activity of brown kidney bean lectin in the presence of the carbohydrate indicates its binding specificity toward the lectin [26].

To determine the minimal concentration of the specific carbohydrate for reduction of hemagglutinating activity of brown kidney bean lectin, the lectin was dissolved in solutions containing different concentrations of the specific carbohydrate in PBS. The hemagglutinating activity assay was performed using the carbohydrate solutions of their particular concentrations for serial two-fold dilution instead of PBS [26].

### Assay of Mitogenic Activity

Splenocytes were isolated from BALB/c mice (body weight: 20 to 25 g, age: 4 to 6 weeks) and were adjusted to a cell density of 2×10^6^ cells/ml. Splenocytes (100 µl) were added to each of the wells of a 96-well plate, followed by 100 µl of different concentrations of brown kidney bean lectin (0 µM–16.2 µM). The cells were incubated at 37°C in a humidified atmosphere of 5% CO_2_ for 48 hours. (Methyl-^3^H)-thymidine (0.25 µCi, GE Healthcare) (10 µl) was added to the splenocytes, and the cells were incubated for another 24 hours under the same conditions. The splenocytes were harvested onto a glass fiber filter. The radioactivity was measured using a Topcount liquid scintillation counter [27].

*The protocol of the study was approved by Animal Experimentation Ethics Committee, The Chinese University of Hong Kong. Permit number: 11–308 DH/HA%P/8/2/1 (19). The animals were handled by experienced researchers, suffering of the animals was minimized.

### Assay of Cytokine Inducing Activity

Splenocytes were isolated from BALB/c mice (body weight: 20 to 25 g, age: 4 to 6 weeks) and were added to 90 mm dishes. Different concentrations of brown kidney bean lectin (0 µM, 0.5 µM and 2.5 µM) were added to the dishes. The cells were incubated at 37°C in a humidified atmosphere of 5% CO_2_ for 4 hours [28].

TRIZOL® reagent (Invitrogen) (1.5 ml) was added to the splenocytes and incubated at 4°C for 5 minutes. Then, 0.3 ml of chloroform was added, followed by vigorous shaking of the mixture. The mixture was centrifuged at 20000 *g* for 15 minutes at 4°C. The upper aqueous layer was transferred to a fresh microtube. 0.5 ml isopropanol was added to precipitate the RNA. The RNA pellet was washed with 75% ethanol, and resuspended in diethyl pyrocarbonate (DEPC)-treated water [28].

The RNA extracted from the mouse splenocytes was converted into cDNA by reverse transcription (RT), using the GeneAmp® RNA PCR kit from Applied Biosystems Company. One µg of RNA was used to yield a 20 µl reaction mixture containing PCR buffer II, 5 mM MgCl_2_, 1 U/µl RNAse inhibitor, oligo dT_16_ and 1 mM dNTP, 2.5 U/µl murine leukemia virus (MuLV) reverse transcriptase. The solution was heated at 95°C for 5 minutes and reverse transcribed at 42°C for 1 hour in a GeneAMP PCR System 9600 (Applied Biosystmes Company) [28].

Polymerase chain reaction (PCR) was performed using the AmpliTag® Gold kit from Applied Biosystems Company. RT product (5 µl) was added to a PCR reaction mixture to yield a 20 µl reaction mixture containing gold buffer, 2.5 mM MgCl_2_, Upper and lower primers (0.5 µM each) (Table 1), 200 µM dNTP and 1.25 U AmpliTag® Gold. PCR was performed for 25 cycles. The PCR products were visualized using agarose gel electrophoresis at 120 V for 30 minutes in tris-borate-EDTA (TBE) buffer, pH 8.3, using 1.5% gel with 0.5 µg/ml ethidium bromide [28].

*The protocol of the study was approved by Animal Experimentation Ethics Committee, The Chinese University of Hong Kong. Permit number: 11–308 DH/HA%P/8/2/1 (19). The animals were handled by experienced researchers, suffering of the animals was minimized.

### Assay of Anti-proliferation Activity

Human breast cancer (MCF7), hepatoma (HepG2), nasopharyngeal carcinoma (CNE1 and CNE2) and normal nasopharyngeal (NP69) cells from American Type Culture Collection were adjusted to a cell density of 5×10^4^ cells/ml in RPMI medium. The cells (100 µl) were seeded onto the wells of a 96-well plate and incubated overnight. Different concentrations of brown kidney bean lectin (100 µl, final concentrations at 0 µM–48 µM) were added to the wells followed by incubation for 24 or 48 hours. After incubation, the medium was discarded, and the wells were washed with phosphate buffered saline (PBS). Then, 25 µl of a 5 mg/ml solution of 3-(4, 5-dimethylthiazol-2-yl)-2, 5-diphenyltetrazolium bromide (MTT) in PBS was added into the wells and were incubated for 4 hours. The plates were centrifuged at 3600 *g* for 5 minutes and the supernatant was carefully removed, and 150 µl of dimethyl sulfoxide (DMSO) was added into the wells to dissolve the MTT (formazan) in the wells. OD 590 nm was measured with a microplate reader within 10 minutes. Percentage inhibition of the MCF7, HepG2, CNE1, CNE2 and NP69 cells was calculated by: [(OD 590 nm of the control – OD 590 nm of a culture exposed to a particular lectin concentration)/OD 590 nm of the control] × 100% [14].

### Annexin-V and Propidium Iodide (PI) Staining

MCF7 cells (5×10^5^) were treated with different concentrations of brown kidney bean lectin (0 µM–36 µM) on 6-well culture plates for 24 hours. The cells were trypsinized and were centrifuged down at 2000 *g* for 4 minutes. Then, the cells were washed with PBS and centrifuged at 2000 *g* for 4 minutes, and repeated three times. The cell pellets were resuspended in 250 µl binding buffer (0.01 M HEPES, pH 7.4, containing 140 mM NaCl and 25 mM CaCl_2_), and 2.5 µl Annexin V-FITC (5 mg/ml) solution (BD Phamingen, CA, USA) and 0.5 µl PI (6 mg/ml) (Sigma) were added. The cells were incubated at room temperature for 20 minutes in dark. The cells were analyzed using a FACSort flow cytometer (Becton Dickinson, Cowley, UK). The signal was detected by FL-1 (530 nm) channel and data analysis was conducted by using the program WinMDI (Version 2.8, Joseph Trotter, La Jolla, CA, USA) [29].

### Measurement of Mitochondrial Transmembrane Potential by JC-1 Staining

MCF7 cells (5×10^5^) were seeded onto a 6-well culture plate and incubated overnight. Different concentrations of brown kidney bean lectin (0 µM–36 µM) were added to the cells and the cells were incubated for 24 hours. The cells were trypsinized and centrifuged down at 2000 *g* for 4 minutes. Then, the cells were washed with PBS and centrifuged at 2000 *g* for 4 minutes three times. The cell pellets were resuspended in RPMI medium (500 µl), containing 2.5 µg/ml JC-1 dye, and incubated at 37°C in the dark. The cells were analyzed using a FACSort flow cytometer for detecting the mitochondrial depolarization patterns [30].

### Cell Cycle Analysis

MCF7 cells (2×10^6^) were treated with different concentrations of brown kidney bean lectin (0 µM–36 µM) in 90 mm culture dishes for 24 hours. The cells were trypsinized and spun down at 2000 *g* for 4 minutes. Then, the cells were washed with PBS and centrifuged at 2000 *g* for 4 minutes three times. One ml of 75% ethanol was added to the cells which were kept at –20°C for 2 hours. The cells were centrifuged down at 2000 *g* for 4 minutes and were washed with PBS three times. The cell pellets were resuspended in 250 µl PBS containing 5 µl PI (6 mg/ml) (Sigma). The cells were incubated at room temperature for 20 minutes in dark. The cells were analyzed using a FACSort flow cytometer (Becton Dickinson, Cowley, UK) [31].

### Hoechst 33342 Staining

MCF7 and CNE2 cells (2×10^5^) were seeded onto 12-well culture plates and incubated overnight. Different concentrations of brown kidney bean lectin (0 µM–12 µM) were added to the cells and the MCF7 and CNE2 cells were incubated for 24 hours and 48 hours, respectively. The medium was discarded, and the cells were washed with PBS three times. Hoechst 33342 in PBS (1 µM) was added to the cells followed by incubation at room temperature for 10 minutes in the dark. The cells were observed under ultraviolet illumination with a NIKON TE2000 microscope (Nikon, Japan) [32].

### Western Blotting

MCF7 cells (1.5×10^6^) were treated with different concentrations of brown kidney bean lectin (0 µM–15 µM) for 24 hours. Proteins from the cells were extracted using RIPA buffer. SDS-PAGE was performed using a 15% separating gel and a 5% stacking gel at a constant voltage of 120 V. After electrophoresis, the proteins were transferred onto a PVDF membrane using Trans-Blot® SD Semi-Dry Electrophoretic Transfer Cell (Bio-Rad) at a constant voltage of 15 V for 45 minutes. The membrane was blocked with 5% non-fat milk in TBST, followed by incubation with primary and secondary antibodies (Cell Signaling), and visualized by ECL detection system (Bio-Rad) [33]. The primary antibodies used include: rabbit β-actin antibody, rabbit caspase 12 antibody, rabbit caspase 9 (human specific) antibody, rabbit IRE-1α antibody and mouse CHOP-antibody, all at 1∶1000 dilutions. The secondary antibodies used include: anti-rabbit IgG, HRP-linked antibody and anti-mouse IgG, HRP-linked antibody, all at 1∶1000 dilutions.

## Results

A 2-step purification enabled acquisition of brown kidney bean lectin (BKBL) with satisfactory purity from a crude extract of the beans. This involved collection of the fraction (fraction II in Fig. 1A) adsorbed on Affi-gel blue gel, followed by the major peak (fraction III in Fig. 1B) from Superdex FPLC-gel filtration column. The first chromatographic step on Affi-gel blue gel helped to remove most of the impurities in the crude extract, allowing BKBL to remain as the major protein in the adsorbed fraction (Fig. 2B). This step led to purification of BKBL with about 13.3 folds (Table 2). The second step, FPLC-gel filtration on Superdex 75 10/300 GL column, helped to remove the remaining impurities to yield purified BKBL (Fig. 2C). The 2-step purification protocol facilitated simple and efficient purification of BKBL with a high yield and protein recovery.

BKBL showed a 32-kDa single band in SDS-PAGE (Fig. 2C). It was eluted in about the 10^th^ ml in FPLC-gel filtration on a Superdex 75 10/300 GL column. Based on the calibration curve of the column, BKBL had a molecular size of around 60 kDa. This showed that BKBL is a 64-kDa dimeric protein consisting of two 32-kDa subunits.

BKBL had a moderate thermostability and pH stability. Hemagglutinating activity of BKBL was completely reserved at 60°C, but further increase in temperature caused abrupt reduction in the activity (Fig. 3A). Also, full hemagglutinating activity of BKBL was observed at pH 3–12, but only half activity remained at pH 0–2, and only residual activity was present at pH 13–14 (Fig. 3B). BKBL was a glucosamine binding lectin. The presence of 250 mM glucosamine reduced its hemagglutinating activity to 25% (Table 3). It was observed that 12.5 mM glucosamine was the minimal concentration required to show reduction in the hemagglutinating activity of BKBL (Table 3).

BKBL displayed the N-terminal amino acid sequence ANEEYFDFQQ (Table 4). Compared to other *P. vulgaris* lectins, BKBL exhibited about 50–60% homology to their N-terminal amino acid sequences. The percentage homology was lower (30–50%) compared with lectins from other *Phaseolus* species.

BKBL exerted immunomodulatory effects. It evoked a mitogenic response from murine splenocytes. In the assay of mitogenic activity, concanavalin A (Con A) was used as the positive control (Fig. 4A). Upon treatment with 0.25–8 µM BKBL, the (methyl-^3^H)-thymidine uptake of murine splenocytes was enhanced (p<0.05), indicating an increase in DNA synthesis and proliferation of the splenocytes (Fig. 4B). The mitogenic response was maximal at a BKBL concentration of 2.5 µM. However, in the presence of 250 mM glucosamine, the specific binding carbohydrate of BKBL, the BKBL-induced mitogenic response was attenuated. BKBL augmented the expression of some cytokines (Fig. 5). In the presence of 0.5 µM BKBL, the mRNA levels of IFN-γ, IL-2 and TNF-α were up-regulated, and a stronger up-regulation of those cytokines was detected under treatment with 2.5 µM BKBL.

BKBL exerted anti-proliferative effects on some tumor cell lines. Based on the MTT assay, after 24-hour treatment with BKBL (Fig. 6A), viability of CNE1 and MCF7 cells was greatly reduced, with an IC_50_ of 3.12 µM and 5.12 µM, respectively. The anti-proliferative activity toward HepG2 and CNE2 cells was comparatively weaker; the IC_50_ values were 32.85 µM and 40.12 µM, respectively. On the other hand, BKBL only slightly inhibited proliferation of normal NP69 cells. When the treatment with BKBL was lengthened to 48 hours (Fig. 6B), the anti-proliferative effects of BKBL were more pronounced. While the IC_50_ value toward MCF7 cells was slightly reduced to 4.80 µM, the anti-proliferative activity on HepG2 and CNE2 became much more conspicuous, with the IC_50_ values reduced to 8.45 µM and 6.64 µM, respectively. However, normal NP69 cells were more responsive to BKBL with an IC_50_ of 16.70 µM, yet the effect was still much weaker than that on the tumor cell lines.

Besides, the effect of glucosamine on anti-proliferative activity of BKBL was tested (Fig. 7). The presence of 100 mM glucosamine had slightly reduced the viability of the MCF7 cells. However, 100 mM glucosamine reduced the anti-proliferative effect on MCF7 cells induced by BKBL, causing the IC_50_ to be raised from 5.12 µM to 21.41 µM. This showed that the anti-proliferative activity of BKBL was based on its carbohydrate binding capability, and the glucosamine added competed for binding sites on BKBL, thus reducing the anti-proliferative effects on the cell lines.

Flow cytometry was used for further investigations of BKBL-induced anti-proliferative activity. In Annexin V-FITC and PI staining of BKBL-treated MCF7 cells (Fig. 8A), the majority of the cells at the lower left quadrant had gradually shifted to the right as BKBL concentration increased, indicated increased phosphatidyl serine (PS) externalization. These were the cells entering the early apoptosis phase. Besides, the proportion of cells at the upper right quadrant also increased with an escalation of BKBL concentration, indicating more cells entering the late apoptosis phase and dying. In JC-1 staining of BKBL-treated MCF7 cells (Fig. 8B), the increase in BKBL concentration caused the majority of the cells to shift from upper left quadrant toward the lower right quadrant, indicating more cells were experiencing mitochondrial depolarization and undergoing cell death. In cell cycle analysis (Fig. 8C), after treatment with low concentrations of BKBL (0.44 µM, 1.33 µM), there was a mild increase in percentage of MCF7 cells in G_2_ phase, while that in G_0_/G_1_ phase decreased. There was a mild cell cycle arrest in G_2_ phase in MCF7 cells at low BKBL concentrations. However, when BKBL concentration was further increased, the percentage of MCF7 cells with damaged DNA had greatly increased. This signified significant damage of MCF7 cells at high BKBL concentrations.

When the MCF7 and CNE2 cells had been treated with 4 µM and 12 µM BKBL for 24 and 48 hours respectively, significant morphological changes (shrinking, losing adherence) of the cells could be observed under a microscope (Fig. 9). With Hoechst 33342 staining, apoptotic bodies (labeled with asterisks) and condensed chromatin (labeled with arrows) could be visualized. Treatment with 4 µM and 12 µM BKBL induced cell death in MCF7 and CNE2 cells by promoting DNA fragmentation and formation of apoptotic bodies.

In Western blotting of MCF7 cells, the increase in BKBL concentration reduced the protein level of pro-caspase 12, while it elevated the levels of cleaved (active) caspase 12, caspase 9, IRE-1α and CHOP (Fig. 10). Caspase 12, IRE-1α and CHOP were the components involved in endoplasmic reticulum (ER) stress. This showed BKBL initiated the cell death of MCF7 cells through ER stress.

## Discussion

Brown kidney bean lectin (BKBL) was purified from the bean extract after only two simple chromatographic steps. Compared with purification of most *P. vulgaris* lectins that required more chromatographic steps, the preparation of purified BKBL was simpler and more convenient. With the use of fewer chromatographic steps, the loss of BKBL throughout the purification process could also be reduced, thus the yield of BKBL could be improved. For 100 g of brown kidney beans, 428 mg BKBL were obtained, with about 57% recovery (Table 2), compared with French beans (cultivar no. 1) which only gave 96.25 mg purified lectin per 100 g beans with 18.73% recovery [12], French beans (cultivar no. 12) with 4.8 mg purified lectin per 100 g beans with 18.1% recovery [34], and Hokkaido red beans with 24 mg purified lectin per 100 g beans with 17% recovery [35].

BKBL is a glucosamine specific lectin. Glucosamine could act as a competitor for binding sites of BKBL, thus reducing the activity of BKBL toward its targets. From the above experiments, the hemagglutinating activity, as well as mitogenic activity and anti-proliferative activity of BKBL, was attenuated upon co-treatment with glucosamine. This showed that these activities of BKBL were dependent on its carbohydrate binding activity. The lectin-carbohydrate binding is reversible: the activity of lectin can be inhibited or recovered by application or removal of the specific carbohydrate, thus providing a good way for controlling the activity of BKBL.

Lectins can be classified according to their carbohydrate binding specificity, such as mannose-binding, glucose-binding, galactose-binding and N-acetylgalactosamine-binding [36]. As a species that is grown worldwide, *P. vulgaris* represents a large group of cultivars. Different cultivars may produce different lectins, with a variety of carbohydrate specificity. Lectins from two cultivars of red kidney bean are mannose-binding and N-acetyl-D-galactosamine-binding respectively [37], [38]. Lectin from Bilozema bean is galactose- and N-acetyl-D-galactosamine-binding [39]. Lectin from French bean cultivar no. 1 is glucuronic acid binding [12]. Some *P. vulgaris* lectins like the lectins from RAZ-2 bean, Tora bean and great northern bean bind to complex β-glucans and N-linked oligosaccharides, instead of simple sugars [40]–[42]. Although *P. vulgaris* lectins can interact with a variety of carbohydrates, none of them but BKBL was found to be glucosamine-specific. This enables BKBL to recognize and bind to glucosamine-containing targets that other *P. vulgaris* lectins cannot.

BKBL induced mitogenic response in murine splenocytes, with maximal activity at 2.5 µM. This matched the results of cytokine induction assay demonstrating that treatment with 2.5 µM BKBL had stimulated murine splenocytes to produce higher levels of interleukin-2 (IL-2), interferon-γ (IFN-γ) and tumor necrosis factor-α (TNF-α) than the 0.5 µM treatment. IL-2 is involved in T-cell activation, while IFN-γ is involved in macrophage activation, increase of expression of MHC molecules and antigen processing components, promoting cellular immunity while suppressing humoral response. TNF-α is involved in local inflammation and endothelial activation, by inducing acute-phase response that promotes the secretion of pathogen binding molecules (e.g. mannan-binding lectin), and inducing fever that can reduce bacterial and viral growth.

BKBL induced anti-proliferative activity toward several tumor cell lines (MCF7, HepG2, CNE1 and CNE2). Flow cytometry studies and Hoechst 33342 staining suggested that apoptosis was induced in MCF7 cells. For Annexin V-PI staining, the right-shifting of the cells indicated phosphatidyl serine (PS) externalization which is a characteristic of early apoptosis. In JC-1 staining, BKBL dose-dependent shifting indicated mitochondrial membrane disruption, which is often involved in apoptosis. DNA condensation and the formation of apoptotic bodies in BKBL-treated cells were observed with Hoechst 33342. Normal cells have their circular nuclei located in the middle of the cells, with the chromatin evenly distributed. When a cell undergoes apoptosis, the nucleus will move toward the edge of the cell, with disruption of its circular structure (becoming U-shape). Close packing of DNA also caused intense Hoechst 33342 staining. Apoptotic bodies were also formed for DNA degradation. The nucleus split into small vesicles, which could be visualized as a group of small spots in Hoechst 33342 staining. DNA condensation and formation of apoptotic bodies are distinct features in apoptosis.

Western blotting disclosed that BKBL induced ER stress in MCF7 cells. Upon accumulation of misfolding proteins, the proper functioning of ER will be threatened, resulting in initiation of unfolded protein response (UPR). UPR will lead to cell cycle arrest and translation attenuation to allow clearance of the unfolded proteins. However, strong or prolonged ER stress can trigger apoptosis. Some signaling pathways are involved in UPR. First, PERK will be activated by autophosphorylation. Then it activates eIF2 to enhance expression of CHOP, which down-regulates the anti-apoptotic mitochondrial protein Bcl-2. This results in mitochondrial damage and apoptosis. Second, IRE-1α will be activated and up-regulated. This activates TRAF2, which in turn triggers the JNK signaling pathway, resulting in apoptosis. Third, ATF6 will be activated, which act as a transcription factor to regulate gene expression. Besides, ER stress also causes Ca^2+^ efflux from the ER lumen. This activates pro-caspase 12, triggering the caspase cascade. The up-regulation of IRE-1α, CHOP and activation of caspase 12 provides evidence that BKBL exerts ER stress, contributing to apoptosis of the MCF7 cells.

There were a number of reports on the mechanisms of anti-proliferative effects of different lectins. Concanavalin A (Con A) was internalized into the tumor cells and accumulated in mitochondria, followed by alteration of the PI3k-Akt anti-apoptotic pathway by downregulation of phosphorylated Akt in HepG2 cells [43]. *Polygonatum cyrtonema* lectin was involved in regulation of Bax, Bcl-XL and Bcl-2 proteins, collapsing the mitochondrial membrane potential, inducing apoptosis through mitochondria-mediated ROS-p38-p53 pathway in A375 cells [44], [45]. Lectin from French bean cultivar no. 35 (*P. vulgaris*) was found to increase Fas expression, followed by caspase 8 activation and mitochondrial membrane potential disruption in MCF7 cells [14]. Previously there were no reports on plant lectin-induced apoptosis through ER stress and UPR. Here, we demonstrated that BKBL had induced up-regulation of IRE-1α, CHOP and activation of caspase 12, which are the distinct changes upon UPR. The BKBL-induced UPR resulted in apoptosis of the MCF7 cells.

BKBL manifested only some homology in N-terminal amino acid sequence with other *P. vulgaris* lectins ( = <60%) as well as lectins from other *Phaseolus* species ( = <50%) (Table 4). The differences in amino acid sequence may explain the differences in their protein structures, as well as differences in their anti-proliferative effects on tumor cells. Although UPR was found to be involved in BKBL-induced apoptosis in MCF7 cells, further studies are needed to see whether other apoptotic pathways such as mitochondrial pathway are also involved.

BKBL acted differently toward different cell types. It evoked mitogenic response of murine splenocytes at 0.25–8 µM, while it exhibited anti-proliferative activity on the tumor cell lines. The IC_50_ of MCF7 cells (5.12 µM for 24 hours, 4.80 µM for 48 hours), CNE1 cells (3.12 µM for 24 hours) and CNE2 cells (6.64 µM for 48 hours) had fallen on the same concentration range of the mitogenic response. This signifies that BKBL can initiate its immunomodulatory effects on splenocytes, as well as anti-proliferative effects on tumor cells simultaneously. The two activities may synergize in treatment of cancers. The relatively low toxicity of BKBL toward the normal (NP69) cell line also suggests the potential of BKBL for therapeutic uses.
